# A 54-Year-Old Woman with Donor Cell Origin of Multiple Myeloma after Allogeneic Hematopoietic Stem Cell Transplantation for the Treatment of CML

**DOI:** 10.1155/2016/6751914

**Published:** 2016-02-18

**Authors:** Erika Maestas, Shikha Jain, Patrick Stiff

**Affiliations:** Loyola University Medical Center, 2160 South First Avenue, Maywood, IL 60153, USA

## Abstract

Chronic myeloid leukemia is a myeloproliferative disorder that may be treated with hematopoietic stem cell transplantation (HSCT). While posttransplantation relapse of disease resulting from a failure to eradicate the patient's original leukemia could occur, patients may also rarely develop a secondary malignancy or myelodysplastic syndrome (MDS) of donor origin termed donor cell leukemia (DCL). Cases of donor-derived acute myeloid leukemia (AML) or MDS after HSCT or solid tumor transplantation have been published. However, very few cases of donor-derived multiple myeloma (MM) exist. We describe a patient who developed a donor-derived MM following allogeneic HSCT from a sibling donor.

## 1. Introduction

Chronic myeloid or chronic myelogenous leukemia (CML) is a clonal myeloproliferative disorder that arises from the neoplastic transformation of undifferentiated hematopoietic stem cells [1]. It is the result of a translocation t(9;22)(q34q11), known as the Philadelphia chromosome, leading to a BCR-ABL fusion gene that encodes transcripts and fusion proteins with abnormal tyrosine kinase activity [2].

Tyrosine kinase inhibitors such as imatinib have revolutionized the treatment of CML. However, prior to the approval of this therapy, allogeneic stem cell transplantation was thought to be the only curative treatment for CML [2]. Posttransplantation relapse of disease is possible and likely results from a failure to eradicate the patient's original leukemia. Posttransplantation immunosuppression may furthermore act as a risk factor for malignancy. However, patients may also rarely develop a secondary malignancy or myelodysplastic syndrome of donor origin termed donor cell leukemia (DCL) [3]. The first case of DCL was described by Fialkow et al. in 1971 and it has since mostly been published in case reports with acute myeloid leukemia (AML) being the most commonly described phenotype [4]. Posttransplantation donor-derived multiple myeloma is an exceedingly rare phenotype of DCL that has been reported following solid organ transplantation of the heart, lungs, and kidneys [5–7]. However, to our knowledge only one case of donor-derived multiple myeloma following HSCT for refractory sideroblastic anemia has been published [8]. On our review, no cases have described a donor-derived multiple myeloma following HSCT for CML. In this case report, we describe a patient who developed donor-derived MM following an allogeneic HSCT for CML from a sibling donor.

## 2. Case Presentation

We describe a 54-year-old woman who initially presented in 1999 at the age of 40 with newly diagnosed CML identified on routine blood work during pregnancy. She eventually miscarried. She was initially treated with Hydrea and underwent sibling allogeneic HSCT with her brother as a donor in January 2000. She tolerated the transplant without complications and was in remission until January 2012 when routine blood work identified increased serum protein and anemia. Serum protein electrophoresis (SPEP) demonstrated an abnormal band in the gamma region and M protein spike of 1.9. Urine protein electrophoresis (UPEP) demonstrated a monoclonal kappa light chain. Bone marrow biopsy demonstrated a monoclonal plasmacytosis of 10–12% consistent with plasma cell dyscrasia. There was no evidence of CML. FISH studies and BCR-ABL testing were negative. A bone study was negative for blastic and lytic lesions. Cytogenetics showed a normal male karyotype of donor origin. These findings were consistent with a new diagnosis of IgG kappa smoldering multiple myeloma.

Our patient was followed with watchful waiting until repeat bone marrow biopsy showed multiple myeloma with rapid progression to active disease. FISH study at diagnosis was positive for an IgH/FGFR3 rearrangement. She was treated with Revlimid, Velcade, and dexamethasone beginning in February 2013. She then continued to autologous HSCT with melphalan as a conditioning regimen in July 2013.

Based on the patient's full chimerism, it was recommended that her sibling donor be evaluated. His labs were significant for an elevated serum protein, SPEP with an abnormal band in the gamma region, and an M protein spike of 2.3. Her sibling donor underwent bone marrow biopsy in April 2012 that showed monoclonal plasmacytosis (~20% on CD138 stain) consistent with myeloma. Similar to our patient, his FISH studies were also positive for IgH/FGFR3 rearrangement in 5.5% of cells. Flow cytometric analyses also showed a monoclonal plasma cell population with cytoplasmic kappa light chain restriction. Findings were consistent with IgG kappa smoldering multiple myeloma that also progressed to active disease. He was also treated with Velcade, dexamethasone, and Revlimid before undergoing autologous stem cell transplant in February 2014.

## 3. Discussion

Donor cell leukemia (DCL) is a rare complication of allogeneic stem cell transplantation. A 2011 review of the literature by Wiseman identified 51 cases of suspected or proven DCL and 13 cases of donor cell-derived MDS [9]. Multiple myeloma only makes up 1% of these posttransplant malignant lesions [6]. The most commonly described phenotype of DCL is AML although cases of myeloid sarcoma [3], gingival squamous cell carcinoma [10], B-cell immunoblastic sarcoma [11], and cutaneous melanoma [12] have also been described. Posttransplantation MM has been reported following solid organ transplantation of heart, lung, and kidneys [5, 6]. Only one case to our knowledge has been published documenting donor-derived MM following HSCT. This was in a patient with refractory sideroblastic anemia [8].

While the incidence of recurrent leukemia decreases over time, donor-derived leukemia tends to occur late [13]. A retrospective study by Wang et al. found that the median time to diagnosis of DCL after transplantation was 24 months [14]. The etiology of DCL is likely multifactorial, although the exact pathophysiology is unclear. Hypotheses have proposed that DCL may occur through the unintentional transfer of an occult malignancy from donor to recipient. It has also been suggested that outwardly healthy donor HSCs have an inherited or acquired predisposition to become malignant. Additionally, cytotoxic effects of chemotherapy for primary malignancy may induce DNA damage that leads to leukemogenic transformation of donor cells after transplantation. Other theories include DCL introduced via viral transfection and integration; oncogene integration and fusion of malignant DNA fragments released into donor HSCTs; bystander radiation damage from conditioning total body irradiation that causes release of leukemic DNA leading to transfection and integration into cells; defective marrow stroma microenvironment; and impaired immune surveillance [9, 13]. In our patient, transplant related MM is thought to have occurred from a precursor myeloma cell, which was likely dormant at the time of allogeneic HSCT that may have been inadvertently transferred during the transplant.

Diagnosis of DCL relies on the identification of cytogenetic and molecular differences between the recipient and donor. In early cases, diagnosis of DCL was made through demonstration of a sex mismatch (i.e., presence or absence of a Y chromosome) between a recipient and donor as evidenced by karyotype analysis or through florescent in situ hybridization (FISH) analyses. However, newer quantitative methods of diagnosis have proved to more effectively diagnose DCL through the study of polymorphic DNA sequences. These PCR based tools involve the study of variable nontandem repeats (VNTR), short tandem repeats (STR), and restriction fragment length polymorphism to identify donor/recipient chimerism [9].

The treatment of DCL is dependent on the phenotype of the donor-derived malignancy. In our patient, her donor-derived multiple myeloma was treated with Velcade, Revlimid, and dexamethasone followed by autologous stem cell transplantation.

## 4. Prognosis

The small number of reported cases, variable follow-up, and range of treatment strategies employed limit prognosis in patients.

## 5. Conclusions

DCL is a complication of allogeneic stem cell transplantation with many cases going unidentified or underreported. Though rare, this disease provides an opportunity to study leukemogenesis in vivo. In our patient, FISH analysis identified a de novo MM of donor origin. Cytogenetic studies following bone marrow biopsy confirmed de novo myeloma of donor origin. While several case reports have been published documenting the development of AML and other tumors, few, if any, cases report a de novo MM arising in a patient treated for CML. The identification of DCL is important to recognize as it may affect treatment modalities and patient outcomes.
